# Anthropocene, the plastic age and future perspectives

**DOI:** 10.1002/2211-5463.13122

**Published:** 2021-04-01

**Authors:** Raffaele Porta

**Affiliations:** ^1^ Department of Chemical Sciences University of Naples ‘Federico II’ Italy

**Keywords:** anthropocene, bioplastics, circular bioeconomy, plastic pollution, renewable feedstock

## Abstract

The issue of plastic waste is one of the main topics on the international societal and political agenda since ever‐increasing growth in the quantity of plastic materials produced has gone beyond the ability to manage them effectively at their end‐of‐life. Mostly pushed by social campaigns, an ever‐increasing number of initiatives have been taken by different institutions to reduce the huge amount of plastic waste: first of all, specific legal regulations have been introduced, both to realize effective systems of plastic collection, reuse and recycling, and to outlaw the use of unnecessary disposable one‐use items. However, due to the indisputable advantages derived from the use of such a material, every action of decision makers to limit the production or use of plastics is unavoidably affected by economic evaluations, as well as by the deficiency or drawbacks of alternative materials, rather than by environmental reasons. In the three reviews in this Special ‘In the Limelight’ section, Oliver Bajt, Paola Fabbri *et al*. and Frederic Debeaufort – invited speakers at the Special Session on Science & Society, entitled ‘Plastics: revolution, pollution and substitution’, of the 45th FEBS Congress to be held in Ljubljana, Slovenia, on 3–8 July 2021 – describe in detail the consequences of plastic pollution (Bajt, 2021, *FEBS Open Bio* 11, 954‐966), the complex transition to bioplastics (Degli Esposti *et al*., 2021, *FEBS Open Bio* 11, 967‐983) and the possibility to obtain these innovative biodegradable materials from food and marine waste (Debeaufort 2021, *FEBS Open Bio* 11, 984‐998), respectively. This introductory commentary highlights that, in the frame of the bioeconomy paradigm, not only multidisciplinary but also inter‐ and transdisciplinary research with integrated and multifaceted approaches are needed to produce novel eco‐friendly materials with features similar to those of traditional plastics, as well as with acceptable economic and environmental impact.

## Graphical abstract

In the frame of the bioeconomy paradigm, inter‐ and transdisciplinary research with integrated and multifaceted approaches are needed to produce novel eco‐friendly materials with features similar to those of traditional plastics, as well as with acceptable economic and environmental impact. The bioplastics circular bioeconomy is defined as an economy where the basic building blocks for materials must derive from renewable biological resources.

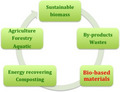

**Abbreviations**
PET: polyethylene terephthalate

## Plastics revolution

**Fig. 1:**
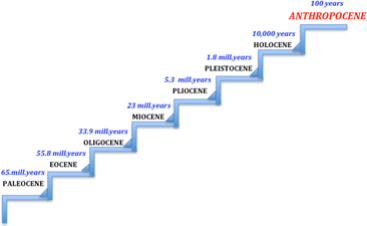
Cenozoic geological timescale.

About 20 years ago, the Nobel Prize‐winning researcher Paul Crutzen was the first to suggest (followed by many other scientists) that we are living in new post‐Holocene Cenozoic era, that he and others dubbed the ‘Anthropocene’, in which humans dominate the Earth's surface geology and ecosystems as never before (Fig. 1) [1, 2, 3]. It is an epoch where everything on the planet is shaped by humans: atmosphere chemical content, free land and forest size, sea levels, climate and, consequently, the number of different living species [4, 5]. Although different start dates for the Anthropocene have been proposed – from the beginning of the agricultural revolution to the explosion of the first atomic bomb – it cannot be disputed that the surface of the planet has been considerably changed from the middle of the last century by the production of a new long‐lasting human‐made material, generally called plastics, that is increasingly leaving an indelible human footprint in lands and sea waters around the world [6]. In fact, various kinds of plastic items are distributed everywhere today, in both terrestrial and marine environments, from the ocean floors to the tops of the mountains, and could be fossilized as a distinctive stratal component in the future. In fact, when future geologists study the Anthropocene, fossilized plastics will be probably considered the key markers of the epoch in which we humans lived. Numerous scientists suggest that the plastic layers are indicative of the start of the Anthropocene and that, after the bronze and iron ages, the current period will be classified as just the ‘plastics age’.

Plastic materials have such a long‐lasting impact on Earth's geology because they are extraordinarily hard to degrade. It is well known that plastic water bottles, for example, take about 500 years to completely disintegrate. But, thanks to its remarkable versatility and utility, this material became crucial for the development of the technological revolution from the start of the so‐called post‐World War II ‘Great Acceleration’ of population, industry and resource utilization. Plastics, produced mostly by oil, first became widespread in the 1940s and then, as a symbol of the definitive transition to modernity, progressively transformed and characterized human life in the form of our cars, trains, airplanes, dresses and sanitary items, and by making packaging easier and helping us to store food for a long time. As an incredibly ductile and versatile material, strong but flexible, light and relatively inert, it has potential to take any form and be available for any use.

**Fig. 2:**
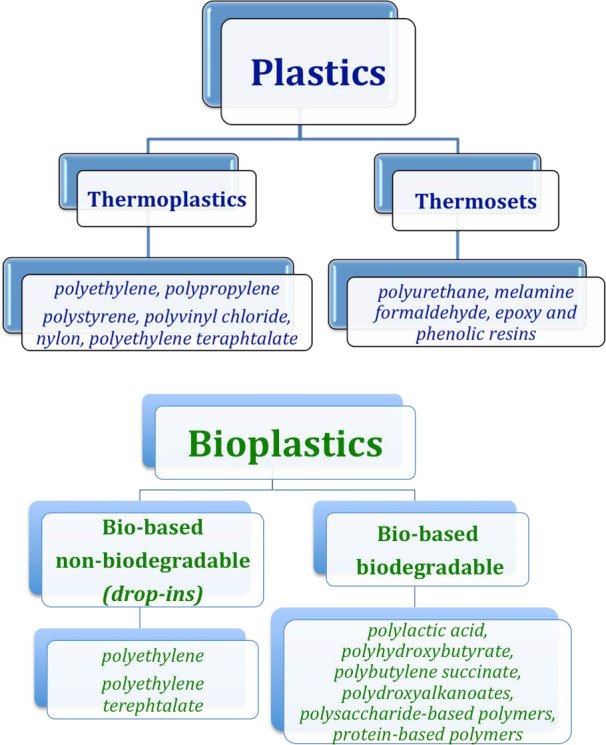
The different types of plastics and bioplastics.

Plastics are easily handleable solids made of various high molecular weight organic polymers. The first man‐made plastics were derived, at the beginning of the 20th century, from natural materials, polysaccharides and proteins (e.g. casein, gelatin, cellulose, rubber), as petrochemical materials were not yet available at that time. But, as early as the middle of the last century, bio‐based plastics had been almost completely replaced by petrochemical plastics. Bakelite, viscose, rayon, nylon, polystyrene, polyvinyl chloride and polyethylene were the first plastic materials to become commonly used [7]. They appeared between the 1920s and the 1940s [8], whereas polypropylene and expanded polystyrene foam were produced in the 1950s, and polyethylene terephthalate (better known as PET), with which most containers are today manufactured, was patented in 1973. The development of new plastics continued until now with the production of over 20 groups of different materials classified into two main subsets: thermoplastics and thermosets (Fig. 2). Thermoplastics, such as polyethylene, polypropylene, polystyrene, polyvinyl chloride, nylon and PET, account for about 90% of the total plastics produced and are chemically stable over a large range of temperatures. These materials can be readily recycled by re‐melting and re‐shaping but, generally, they cannot be mixed together during recycling and, thus, must be separated into the originating monopolymers. Conversely, thermosets, such as polyurethane and melamine formaldehyde, as well as the epoxy and phenolic resins, are polymers characterized by high resistance to mechanical forces, heat and chemicals, and are thus unable to melt and, consequently, more challenging to be reutilized.

## Plastics pollution

Plastics are long‐lived on a human timescale since their degradation takes place very slowly either physically, chemically or biologically. Most effective fragmentation occurs through photodegradation by sunlight ultraviolet rays, whereas chemical degradation results mainly via hydrolysis at extreme pH values, and biological degradation by bacteria, fungi or other multicellular organisms may occur following enzymatic depolymerization [9, 10, 11, 12, 13]. In particular, microbial biodegradation might become more frequent and effective in the future, mostly because the bacteria that become able to use plastics as a food source would be selectively advantaged.

However, the majority of plastic items are designed and manufactured for durability, and it is just this feature that is the cause of the main pollution troubles other than ever‐increasing production rates. The annual output of plastics has in fact gone from less than two million tons in 1950 to over 350 million tons today (about 65 million tons only in the EU), and, if current consumption rates continue, it is expected to be over 30 billion tons by 2050 [14]. There is such a growing amount of plastics on the Earth's surface even in remote environments, such as the deep sea and the polar regions, that plastic material must be considered by now not only as pollutant but, as mentioned above, also as contributor to the characteristics of contemporary strata. Most clearly evident in landfills, low‐density plastics (mainly polyethylene and polypropylene) waste is also visible in floating ‘sea‐fills’, created by the wind and by surface currents and concentrated in mid‐ocean gyres, such as the so‐called *Great Pacific Garbage Patch* of over thousand kilometres diameter [15]. Plastics account for more than 10% of generated total waste and for up to 90% of the marine litter. The amount currently entering the sea water each year ranges from 5 million tons to about 13 million tons, with the amount expected to increase by an order of magnitude by 2025 [16]. A report from the ‘World Economic’ and the ‘Ellen MacArthur Foundation’ predicts that, at this rate, oceans will contain more plastics than fish by 2050 [17]. Finally, most of the plastic materials occurring in the oceans are not visible because primarily physical and mechanical factors, such as sunlight and wave movements, break them into extremely small particles, known as micro‐plastics and nano‐plastics of tens of micrometres and nanometers in diameter, respectively, able to penetrate cell walls and to affect both growth and reproduction of aquatic organisms [18]. However, the technical difficulty of separating these plastic nano‐ and microfragments from the sediment of the sea floor makes it challenging to investigate their distribution.

## Bioplastics strategy

The current and most urgent question is the following: how to counteract this alarming phenomenon? The issue of plastic waste management and consequent terrestrial and marine pollution is top of the political agenda, and there is increasing pressure for businesses and governments to work together to try to tackle this huge concern. The first strategy is to seek to eliminate unnecessary single use and short use phase items that are made wholly or partly made of plastics (e.g. bags, cotton buds, food and drink containers) and make all other plastic products of medium/long phase use (e.g. toys, pipings, window frames) reusable and/or recyclable. To this aim, an effective solid waste separate collection system represents an essential step. Plastics collection in Europe reached almost 27 million tons (about 30% of the total plastic waste) in 2018, 1/3 of which were recycled, 1/4 ended up in landfills and the remaining was combusted in incineration plants [19].

The reduction of total plastic production and the substitution of oil‐derived plastics (mostly those currently constituting single or short use phase items) with alternative biodegradable materials represents a further strategy. Thus, bio‐based plastics were rediscovered and have undergone a significant revival attracting the attention and interest not only of public opinion and institutions, but also of industrial sectors and scientific research. Bio‐based materials (which are not necessarily biodegradable) are products wholly or partly derived from molecules of biological origin, whereas biodegradable ones are those broken down by micro‐organisms giving rise to carbon dioxide and water in aerobiosis or to methane in anaerobiosis (Fig. 2). Biodegradable plastics are considered compostable when they are biodegraded under defined standard conditions, such as those described in the European Standard EU13432. Unfortunately, insufficient research has been carried out thus far in this field, since all the produced bioplastics currently make up only a small proportion of plastics overall. Therefore, further efforts will be needed both to produce novel biodegradable materials with features close to those of traditional plastics and to assess their operational, economic and environmental impact.

The complexity of the challenge posed by the current uncontrolled use of plastics does not allow over‐simplification of issues, particularly if the specific interests of all the stakeholders involved are not recognized. Just consider that the European plastic industry alone, including plastic raw material producers, plastic converters and plastic machinery manufacturers, employs more than one and a half million direct employments distributed among more than sixty thousand companies, all over the EU member states, with a turnover of almost four hundred billion Euros in 2018. This sector is part of one of the most innovative sectors in the EU, with plastics the topic of one in every 25 patents submitted between 2002 and 2013 [19]. For its part, research should highlight the need to take actions which are multi‐, inter‐ and transdisciplinary, as well as specific towards each single problem, by considering the uniqueness of function and the associated environmental impacts of each plastic. In this scenario, it is therefore critical to design a clear roadmap for bioplastics. Some innovative materials have of course potential to provide solutions in different sectors, but their desired role and effectiveness need to be clearly articulated with tailored studies to improve their properties and performance, in order to propose alternatives to specific traditional plastics used for well determined applications.

In recent years, however, several different kinds of novel bioplastics have emerged which aim to enter the plastics market. Total bioplastics production, reported to be about 19 million tons, currently has a share of about 6% of the global plastics market, and it is expected to reach a maximum of 10% of the global market for plastics within the next 5 years [20]. In particular, bio‐based/nonbiodegradable plastics are reported to have increased in the last 5 years from 63% to 82% of total bioplastics production and bio‐PET output alone passed from 41% of total bioplastics to 76% [20].

## Agri‐food renewable feedstock

**Fig. 3:**
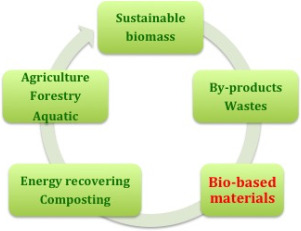
Bioeconomy paradigm for sustainable development.

Increasing biotechnological knowledge is fertilizing the polymer field, enabling the development of novel biopolymers from renewable feedstock. In fact, since several bio‐based plastics require land for their production, raising concerns over the competition for food cultivation, there is growing attention towards a new generation of bioplastics produced from feedstock, such as agri‐food and marine waste or by‐products of their industries, that do not compete with the food chain. These innovative bio‐based biodegradable plastics therefore have the potential to at least partially change the philosophy that organic waste should necessarily be processed in composting facilities, finding possible applications for them in the fight against plastic pollution. The principle of using organic waste and by‐products as bio‐based resources to start producing novel bioplastic materials is a really attractive perspective, because the sustainable bio‐based feedstock might be in theory any biological matter dross, originating from wood, crops or even food waste. This perspective is fully in line with the circular bioeconomy concept of keeping resources in a constant loop, minimizing waste and reducing the need for new feedstock (Fig. 3).

## Bioplastics circular bioeconomy

The emerging bioeconomic paradigm asserts the role of technological innovation in capturing the latent value of renewable bio‐resources to create sustainable growth and development, under which the revitalization of all economic systems could rapidly spread globally [21]. The industrial revolution, oil‐based in the 19th and technology driven in the 20th century, should give way in the current century to a transition inspired by biological resources, in the name of sustainable development of the world economy, aimed at a civilization more considerate of the equilibrium with nature. The bioeconomy is made possible by the continuing advance in scientific knowledge and technical competencies that can be used to exploit numerous biological processes for practical applications. Advances in biosciences, and in particular in biotechnology, are being increasingly used to develop products and processes for high value market applications, and perspectives are very broad, from food and pharmaceuticals to polymers and new biomaterials. Europe's bioeconomy alone is currently worth more than two trillion euros and employs almost 19 million people [22, 23].

The bioplastics circular bioeconomy, defined as an economy where the basic building blocks for materials must derive from renewable biological resources, is increasingly becoming a crucial component in the drive to create a fully sustainable economy. In this respect, by 2021 it is expected that Europe will possess around a quarter of the world’s bioplastics production capacity. However, while the market potential for environmentally friendly alternatives to oil‐based plastics would be extremely wide, the cost of bioplastics still remains a critical factor. To become commercially competitive, one way is to make use of existing waste streams. Therefore, even though the early use of the term bioeconomy referred to any use of biological knowledge for commercial and industrial purposes, today the bioplastics are assuming a pivotal role, not only because of the goal of obtaining maximum value from biological resources, but also because they contribute to a shift away from the circular economy of traditional plastics [24, 25]. Nowadays, one of the main challenges is to use agri‐food and marine residues, as well as other industrial by‐products and carbon‐rich waste streams without market value, for the development of high‐quality bioplastics that can be managed at the end of their life as organic waste by industrial composting. This strategy has the potential to open up new sustainable business opportunities and to hopefully contribute to Europe’s transition towards a postcarbon economy.

## Conflict of interest

The author declares no conflict of interest.

## Author contribution

RP wrote the manuscript and prepared the figures.
